# Early-childhood housing mobility and subsequent PTSD in adolescence: a Moving to Opportunity reanalysis

**DOI:** 10.12688/f1000research.8753.1

**Published:** 2016-05-27

**Authors:** David C. Norris, Andrew Wilson

**Affiliations:** 1 David Norris Consulting, LLC, Seattle, WA, 98102-5384, USA; 2 College of Nursing, University of Utah Health Sciences Center, Salt Lake City, UT, 84132, USA; 3 Anolinx LLC, Bountiful, UT, 84010, USA

**Keywords:** mental health, housing mobility, adolescent, post traumatic stress disorder

## Abstract

In a 2014 report on adolescent mental health outcomes in the Moving to Opportunity for Fair Housing Demonstration (MTO), Kessler et al. reported that, at 10- to 15-year follow-up, boys from households randomized to an experimental housing voucher intervention experienced 12-month prevalence of post-traumatic stress disorder (PTSD) at several times the rate of boys from control households. We reanalyze this finding here, bringing to light a PTSD outcome imputation procedure used in the original analysis, but not described in the study report. By bootstrapping with repeated draws from the frequentist sampling distribution of the imputation model used by Kessler et al., and by varying two pseudorandom number generator seeds that fed their analysis, we account for several purely statistical components of the uncertainty inherent in their imputation procedure. We also discuss other sources of uncertainty in this procedure that were not accessible to a formal reanalysis.

## Introduction

The Moving to Opportunity for Fair Housing Demonstration (MTO) was a social experiment mandated by Congress, and conducted during the 1990’s. From 1994 to 1998, 4,604 households residing in distressed inner-city housing in five U.S. cities were randomized to three experimental groups. A *control* group received no housing voucher; a *traditional voucher* group received a standard ‘Section 8’ housing voucher; and a *low-poverty voucher* group received an experimental type of voucher usable only for housing located in a low-poverty area. Adults and children in the MTO households were surveyed in 2001 and 2011 to assess a variety of economic and mental health outcomes.

A 2003 MTO Interim Evaluation ^1^ (4–7 years after randomization) revealed an interesting interaction between gender and housing mobility, with respect to outcomes of delinquency and risky behavior: girls benefited, but boys did not. This finding, somewhat at odds with earlier research ^2^, spurred subsequent explanatory efforts ^3– 6^.

In 2014, Kessler *et al.* ^7^ published an analysis of the Final Youth Evaluation (10–15 years after randomization), reporting statistically significant and numerically substantial voucher effects on several psychiatric diagnoses in boys. Compared with controls, boys from low-poverty voucher households exhibited elevated 12-month prevalence of PTSD (6.2% vs 1.9%; OR, 3.4 [95% CI, 1.6–7.4]) and ‘conduct disorder’ (6.4% vs 2.1%; OR, 3.1 [95% CI, 1.7–5.8]). One of us (DCN) has previously suggested ^8^ that these results are open to criticism on *construct validity* ^9^ grounds. While working to define a protocol for empirically exploring this question, however, DCN learned that the PTSD outcome in 7 was imputed in a manner that invites scrutiny at the more basic level of *statistical conclusion validity* ^10^.

### PTSD outcome imputation by Kessler *et al.*

Although the report by Kessler *et al.* ^7^ describes in some detail the imputation of missing *covariates* in the Final Youth Evaluation, it does not indicate that its PTSD outcome was in fact imputed. This imputation initially came to attention through a footnote on page 38 of 11, and was subsequently confirmed in a (Nov 2014) communication from the original authors.

The MTO Final Youth Survey employed an abridged, computerized self-administered version of the Composite International Diagnostic Interview (CIDI) ^12^. The full version of the CIDI was used in the National Comorbidity Survey Adolescent Supplement (NCS-A), where its PTSD diagnostic algorithm was found to have a "moderate" concordance (AUC, 0.79) with diagnoses obtained through clinical diagnostic interviews ^13^. The abridgment of the MTO instrument prevented direct application of the CIDI diagnostic algorithms in the MTO, however. Instead, CIDI algorithm-derived lifetime PTSD diagnoses in the NCS Replication survey (NCS-R) were regressed on responses to those PTSD-related questions which were retained in the MTO instrument, and the resulting (logistic) regression model was used to impute PTSD diagnoses for the MTO Final Youth Survey respondents. (In addition to the retained PTSD-related questions, the ‘obligatory’ variables *age, sex* and *race* were also included as regressors; see Table 1). Imputation was performed by Bernoulli draws consistent with the (logit) probabilities yielded by this regression equation. Responses to questions about recency of symptoms were in turn used to compute 12-month prevalence diagnoses from these imputed lifetime diagnoses.

**Table 1.** Coefficients of the logistic regression model used to impute PTSD outcomes in Kessler *et al.* ^7^.

Beta	Independent variable
–1.515	Intercept
0.0263	Age
0.1105	Sex is female (0 = Male, 1 = Female)
–0.0819	Race is hispanic
–0.5597	Race is black
–0.9751	Race is other
–0.5603	as child, badly beaten by parent/caregiver
0.0504	badly beaten by spouse/romantic partner
–0.3877	badly beaten by anyone else
0.1148	mugged/held up/threatened with weapon
–0.1614	ever raped (penetration occurred)
0.5993	ever sexually assaulted or molested
0.078	someone close died unexpectedly
0.4687	anyone close had extreme traumatic exper.
0.4591	as child, witnessed serious physical fights
0.1683	saw person badly injured/killed/dead body
–0.2237	ever experienced other very traumatic event
0.3664	purposely stay away things remind event
–0.0581	lose interest in things used to enjoy
0.2516	feel emotionally distant/cut-off from people
0.1159	trouble feeling love/happiness toward others
0.64	feel no reason to plan for the future
0.8654	trouble falling asleep during random event
0.1323	more easily startled by ordinary noises

### Objections to this imputation procedure

A number of objections to this outcome imputation procedure can be articulated without recourse to a reanalysis.

***The imputation is superfluous***. Viewed as a link in the chain of the analysis, the imputation itself appears superfluous. It introduces an information-destroying and noise-generating transformation of the predictive model’s real-valued outputs—logit probabilities on the continuous interval (–∞, ∞)—to pseudorandom ‘outcomes’ in the set { *no*, *yes*}. In order to interpret conclusions about these imputed outcomes as if they were conclusions about real PTSD, it would be necessary to defend the outputs of the predictive model as genuine probabilities. But if these were true probabilities, then they could be analyzed *directly*, without interposing a noisy and information-destroying pseudorandom number generation (RNG) step.

***It makes the reported CIs strictly artifactual***. Because a logistic regression model has no error term, the confidence intervals reported for the logistic regression of imputed PTSD on voucher treatment in Kessler *et al.* ^7^ convey only the uncertainty arising from the RNG sampling performed to impute the PTSD outcomes. All of the substantive sources of uncertainty lurk in the specification and estimation of the imputation model, procedures that are opaque to a reader of 7.

***The model specification appears desultory***. The specification of this *outcome*-imputation model gives every impression that it was regarded as purely phenomenological, and that it was specified, estimated and checked in the desultory manner customary for imputation of missing *covariates*. The model shows no evidence of an attempt to include additional predictors (beyond the ‘obligatory’ age/sex/race) that the MTO Final Youth Survey shared with NCS-R. No bootstrap-validation is described, nor is any other investigation of the predictive performance of the model in the NCS-R population against which it was estimated. No shrinkage was applied to correct for overfitting ^14^. The model coefficients themselves (see Table 1) seem uninterpretable in causal terms, unless it can be supposed (e.g.) that being badly beaten in childhood confers protection from PTSD in nearly equal measure to the PTSD risk incurred from a sexual assault/molestation.

**Figure 1.**
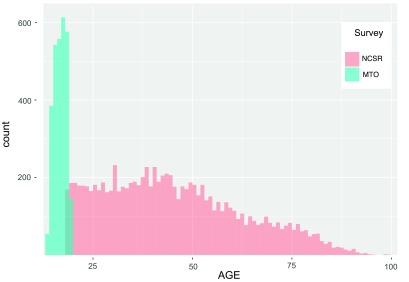
Age distributions of the NCS-R and MTO Youth surveys. The PTSD imputation model used in 7 was estimated in the former population, and applied to the latter.

***Generalizability of NCS-R to MTO is doubtful***. Even if the use of an outcomes-imputation model had been described in 7, and if its performance had been rigorously investigated and overfitting addressed, the question of *generalizability* from NCS-R to MTO populations would still remain open. Most plainly, the NCS-R and MTO Youth populations barely overlap in age ( Figure 1). Also important may be the *qualitative* differences expected between the general-population sample of NCS-R and the inner-city MTO Youth sample, with regard to their traumatic exposures and their sources of resilience relevant to PTSD.

## Methods

To develop a focused critique in an objective form, we undertook a reproduction and reanalysis of the PTSD findings in 7.

In an August 2015 communication to the original authors, we committed to principles of reanalysis articulated by Christakis and Zimmerman ^15^. Of greatest importance, to avoid a “statistical fishing expedition” ^15^, we committed to limiting the scope and methods of our reanalysis to those discussed in an earlier communication (Nov 2014), which is basically recapitulated in points 1–4 above. Our commitment incorporated an *explicit exclusion* of multiple aspects of the original analysis: “We do not intend to critique your multiple imputation of partially missing covariates, nor the weighting techniques you employed, nor your case-level imputation to correct for non-response. We will take these as givens, as manifested in your SAS code and in the precomputed weights in the data” (Aug 2015). Additionally, regarding as too weak Christakis and Zimmerman’s requirement that original authors “should be provided with the opportunity to review and comment on the reanalysis before its acceptance for publication” ^15^, we committed in this same communication also to giving the original authors “access to all of our reanalysis code no later than our manuscript is submitted for peer review.” (Said access was provided on May 3, 2016.) Finally, to avoid publication bias, authors committed to “make all reasonable efforts to publish a manuscript describing our reanalysis findings regardless of the ‘significance level’ of the widened confidence intervals it ultimately yields.”

Our analytic code and results were maintained in a repository on GitHub, with the primary intent to support reproduction and scrutiny of our reanalysis by the original authors, as well as by reviewers and other third parties. All statistical code provided by the original researchers was checked-in to this repository exactly as received, so that any subsequent modification made by us could be readily inspected via the git diff command. A README file in the root directory of this repository provides orientation to directory structure and repository content. A single SAS script was developed which reproduces all steps of our reanalysis.

- We resampled the coefficients of the PTSD imputation model from a multivariate normal distribution with mean and covariance matrix as provided by the original authors in a Jan 2016 communication. The first ‘sample’ was replaced by the coefficients in Table 1, so as to embed a formal reproduction of the original effect estimates within the larger bootstrapping exercise.
- We varied the seed used in generating the pseudorandom *U*(0, 1) probability thresholds necessary for imputing a binary PTSD ‘outcome’ from the (logit) probabilities produced by the PTSD imputation model. In 7, this seed was set to 1234567; we allowed this ‘ pr_seed’ to range over {123, 1234, 12345, 123456, 1234567}.
- We varied the seed used for multiple imputation of missing covariates. In 7, the seed value was 524232; we allowed this ‘ mi_seed’ to range over the 10 consecutive values 524230–524239.

To explore also the impact of arbitrary PTSD model specification, we performed a similar bootstrapping exercise where, instead of resampling the model coefficients, we explored a small set of alternate specifications of the imputation model. A 2×2×2 grid of models was explored in which: (1) *age* was or was not included; (2) *race* was or was not included; and (3) the NCS-R sample was or was not restricted to *age* ≤ 40 before estimating the model coefficients. The eight models thus produced were designated as in Table 2; model specification **a1r1s99** accords with that used in 7.

**Table 2.** Alternative specifications explored for the logistic regression model used to impute PTSD outcomes in Kessler *et al* ^7^. Model ‘a1r1s99’ is the original specification.

Model	*age*	*race*	NCS-R age subset
**a0r0s40**	-	-	≤ 40
**a0r0s99**	-	-	all
**a0r1s40**	-	included	≤ 40
**a0r1s99**	-	included	all
**a1r0s40**	included	-	≤ 40
**a1r0s99**	included	-	all
**a1r1s40**	included	included	≤ 40
**a1r1s99**	included	included	all

Because the standard MTO data package maintained by the Department of Housing and Urban Development (HUD) contains pre-imputed data, whereas the original analysis in 7 employed multiple imputation of the missing values in raw data, a specially prepared MTO data package was required for this work. The National Bureau of Economic Research (NBER) kindly prepared and archived this package with HUD.

## Results

We reproduced the originally reported odds ratios and 95% confidence intervals for voucher effects on 12-month prevalence of PTSD in boys: 3.44025 [1.60147–7.39026] and 2.67817 [1.23268–5.81873] for the low-poverty and traditional vouchers, respectively. (These figures were reported to 1 decimal place in the original article.)

In the course of achieving this reproduction, several unexpected features of the original analysis came to light: (1) the imputation of PTSD outcomes *preceded* the 20× multiple imputation of missing covariates; (2) 24.5% (456/1863) of the MTO boys were *uninterviewed*, and so contributed little more than their baseline characteristics to the analysis.

**Figure 2.**
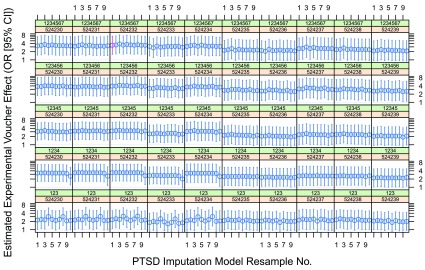
Bootstrapped estimates of the low-poverty voucher effect on PTSD 12-month prevalence in MTO boys. Each panel contains 10 effect estimates obtained by resampling the PTSD imputation model as described in the text, with the first ‘resample’ being the original coefficients as in Table 1. For each panel, the green strip shows the RNG seed used to generate pseudorandom *U*(0, 1) thresholds for PTSD imputation, and the orange strip shows the RNG seed used for the multiple imputation of missing covariates. In 7, these seeds were set to 1234567 and 524232, respectively; thus, the effect drawn in red represents our reproduction of the published effect estimate. Note the logarithmic scale.

Figure 2 shows our 10 × 5 × 10 = 500 bootstrapped estimates of the low-poverty voucher effect on 12-month PTSD prevalence in MTO boys, placing the original estimates (shown in red) in context.

**Figure 3.**
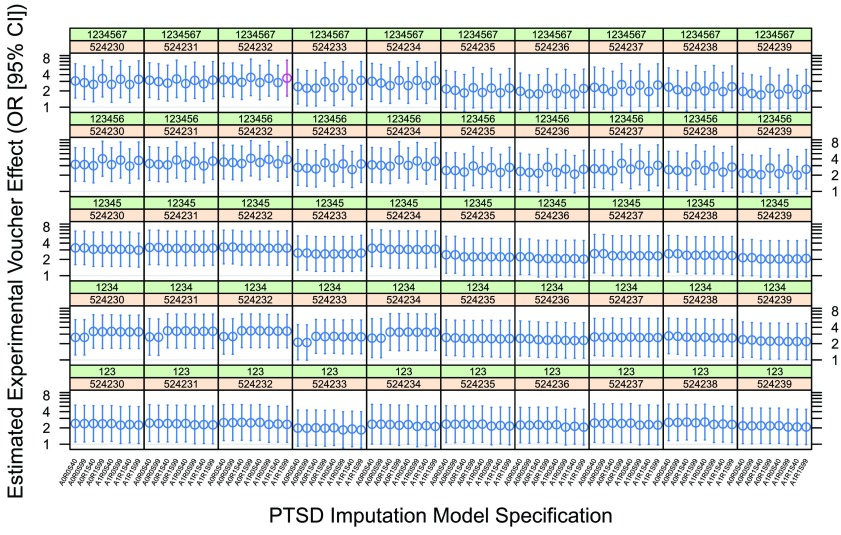
Bootstrapped estimates of the low-poverty voucher effect on PTSD 12-month prevalence in MTO boys. Each panel contains eight effect estimates obtained using the alternative PTSD imputation model specifications in Table 2. For each panel, the green strip shows the RNG seed used to generate pseudorandom *U*(0, 1) thresholds for PTSD imputation, and the orange strip shows the RNG seed used for the multiple imputation of missing covariates. In 7, these seeds were set to 1234567 and 524232, respectively; thus, the effect drawn in red represents our reproduction of the published effect estimate. Note the logarithmic scale.

Figure 3 shows our 8 × 5 × 10 = 400 estimates of the low-poverty voucher effect on 12-month PTSD prevalence in MTO boys, bootstrapped over alternative specifications of the PTSD imputation model.

Each iteration of our bootstrap required just over 4 minutes on a modern Windows workstation; Figure 2 and Figure 3 thus represent about 35 and 28 hours of computation, respectively.

A public fork of our code repository may be accessed at https://github.com/DNC-LLC/MTOpublic.

## Discussion

A social science research enterprise as massive as the MTO Demonstration necessarily involves manifold layers of analysis that may obscure the provenance of analytical results and of associated scientific ‘findings’. This situation creates a compelling rationale for reproduction and reanalysis of such research. The work presented here is, we believe, the first independent attempt to reproduce or reanalyze published results from MTO. (HUD informed us that our March 5, 2015 application for an MTO data license was the first such request received by them.)

A frequentist confidence interval should be understood as abstracting away the arbitrariness immanent in a point estimate, by situating that estimate within the context of an *ensemble* of imagined ‘possible replications’. Regrettably, in much current statistical practice only the arbitrariness of a *sampling procedure* typically is recognized and objectively accounted for 16. Yet other forms of arbitrariness, such as *overfitting* and *model selection*, are equally amenable to similar ‘contextualization’ by bootstrapping techniques such as we employ here. (Indeed, we have structured our reanalysis so as to accentuate this frequentist abstracting-and-contextualizing idiom; our Figure 2 and Figure 3 present a direct visual analogue of the *ensemble*, which we hope will prove broadly accessible to social scientists. A reanalysis performed strictly for a professional statistical audience would dispense with explicit seed variation, instead simply increasing the number of multiple imputations performed from 20 to 1000; it would likewise have done away entirely with the imputation of a binary PTSD outcome, substituting a direct analysis of the PTSD probabilities.)

The confidence intervals reported in 7 account only for the sampling inherent in the MTO design. Our bootstrapping analysis further abstracts away (1) arbitrariness arising when Kessler *et al.* fixed their PTSD imputation model at its maximum-likelihood estimate as if known with infinite precision; (2) the arbitrariness of what appears to have been a desultory model specification; (3) some purely technical forms of arbitrariness appearing in the form of sensitivity to RNG seed selection. While we have achieved only *partial* abstraction of these forms of arbitrariness, and have not addressed *overfitting*, it does appear that the original claim of ‘statistical significance’ for a voucher-on-PTSD effect in MTO boys withstands the formal challenge of our reanalysis as planned. That is to say, a randomly selected confidence interval from Figure 2 or Figure 3 typically sits above the *OR* = 1 threshold.

What is clear enough, however, is that a statistical analysis that randomly selects its ‘findings’ from Figure 2 or Figure 3 cannot be recommended for its wholesome frequentist properties. Indeed, the evident clustering of variance within certain (combinations of) seed choices disallows any straightforward attempt to compute a single, wider confidence interval from these bootstrap samples.

The main contribution of our reanalysis may be simply to bring enough transparency to an otherwise obscure PTSD imputation procedure (1) to avert its application in further research, and (2) to encourage exploration of alternative modes of analysis of the interesting and important social-scientific and policy questions surrounding the MTO boys’ exposures—and responses—to violence.

The close scrutiny involved in a reanalysis of this kind inevitably brings to light aspects of the original analysis which could not have been anticipated, provoking questions not pre-specified as part of the reanalysis. We are now interested to know the statistical impact of including in the original analysis ^7^ (and likewise in the present reanalysis) 456 uninterviewed MTO boys for whom little more than baseline covariates were available—especially in light of the fact that the PTSD imputation *preceded* the multiple imputation of missing covariates. We hope to address this question in a subsequent analysis.

## Conclusions

We have reproduced and reanalyzed a key finding reported by Kessler *et al.* ^7^, following sound principles of reanalysis ^15^ with particular attention to limiting our scope and methodology to issues and techniques proposed before we examined the data. The original claim of a statistically significant effect of the low-poverty voucher on PTSD in adolescent boys has formally withstood the objective challenge presented by this reanalysis. Our reanalysis also brings new transparency to outcomes-imputation methods employed in 7, which may contribute usefully to the evaluation of this work by researchers and policy analysts.

## Data availability

The data referenced by this article are under copyright with the following copyright statement: Copyright: © 2016 Norris DC and Wilson A

*Open Science Framework*: Dataset: Early-childhood housing mobility and subsequent PTSD in adolescence: a Moving to Opportunity reanalysis, doi 10.17605/osf.io/jcpyn ^17^
