# Supplementary material for: Early-childhood housing mobility and subsequent PTSD in adolescence: a Moving to Opportunity reanalysis
Source: F1000Res. 2016 May 27;5:1014. [Version 1] doi: 10.12688/f1000research.8753.1 (PMC4955018; doi:10.12688/f1000research.8753.1)
Supplement: Peer review history

# Peer review: Referee response for version 1

**DOI:** 10.5256/f1000research.9420.r14561

**Published:** 2016-07-15

**Authors:** Fred Hasselman

**Affiliations:** 1 Learning and Plasticity, Behavioral Science Institute, Radboud University Nijmegen, Nijmegen, Netherlands

This article discusses a re-analysis and subsequent re-interpretation of a previously published study on the important topic of PTSD in adolescence. The authors argue the imputation method used in the original article is flawed and I agree with their objections.

 The scientific method is supposed to to be self-correcting; the lucid problem analysis and subsequent solutions presented in this article and decision to disseminate it through a platform like F1000Research are examples of "open science" practices that will enable us to achieve this goal for our discipline.

I have read this submission. I believe that I have an appropriate level of expertise to confirm that it is of an acceptable scientific standard.

# Peer review: Referee response for version 1

**DOI:** 10.5256/f1000research.9420.r14046

**Published:** 2016-06-24

**Authors:** Ewout Steyerberg

**Affiliations:** 1 Department of Public Health, Erasmus University Medical Center, Rotterdam, Netherlands

This is a very timely paper in this era of debate on scientific integrity and transparency. The paper is a good illustration of what can be achieved by data sharing and rigorous re-analysis.

 Specifically, the issues of dealing with missing data are relevant, and especially outcomes-imputation methods. Imputation of missing outcome is anyway controversial; Von Hippel proposed a Multiple Imputation then Deletion (MID) approach, which has some appeal but was not supported by a recent study, which argued that auxiliary variables could be beneficial in this situation to impute missing Y (Sullivan, 2015). The Von Hippel and Sullivan papers need to be discussed briefly as educational efforts in the current paper, in line with the paragraph: ***The imputation is superfluous.***

 There is 2 main issues in the specific hypothesis studied related to housing vouchers: effect estimate and uncertainty in the effect.

1. The MTO project had a strong design, with randomization to 3 groups for evaluation of the intervention. The specific outcomes highlighted here are psychiatric diagnoses, among which PTSD; this was one of many outcomes evaluated perhaps, and not the primary outcome of this study. This brings in a multiple comparisons problem, which may be emphasized more by the current authors.
  We now focus on one association that stood out among many associations that were probably evaluated. First we may wonder whether we should adjust formally for multiple testing, such as a classical Bonferroni correction for the p-value. Second, we know that the strength of the association will be overestimated, even if a true association.
  The suggested boys vs girls interaction further complicates the multiple comparison issue; essentially all analyses are doubled.  I would like some further discussion on these broader issues of interpreting research findings (Ioannadis, 2014).
2. Uncertainty is usually expressed as a 95% confidence interval; the current study appropriately highlights that multiple sources of uncertainty may need to be considered, specifically in the context of multiple imputation.

Overall, this paper is an interesting contribution to statistical aspects on modern analysis. It illustrates that different researchers may use different methods and approaches. It also shows some progress in scientific methods, e.g. the use of bootstrapping is now more feasible than some years ago due to ever increasing computational power.  This type of reanalysis is to be applauded and stimulated to increase the credibility of scientific research findings.

I have read this submission. I believe that I have an appropriate level of expertise to confirm that it is of an acceptable scientific standard.

## References

[1] SullivanTRSalterABRyanPLeeKJ: Bias and Precision of the. *Am J Epidemiol*.2015;182(6) : 10.1093/aje/kwv100 528-34 10.1093/aje/kwv100 26337075

[2] IoannidisJP: How to make more published research true. *PLoS Med*.2014;11(10) : 10.1371/journal.pmed.1001747 e1001747 10.1371/journal.pmed.1001747 25334033PMC4204808
